# Emotional Intelligence in Physical Education in Primary Education: A Systematic Review

**DOI:** 10.3390/healthcare13233166

**Published:** 2025-12-03

**Authors:** José Luis Murillo-Pulido, Flavia Estefanía Amar-Cantos, María Dolores Aguilar-Herrero, Ana Rodríguez-Cano, José Manuel Armada-Crespo

**Affiliations:** 1 Faculty of Education Sciences and Psychology, University of Cordoba, 14071 Cordoba, Spain; 2 Research Group in Sport and Physical Education for Personal and Social Development (GIDEPSO), Faculty of Education Sciences, University of Cádiz, 11519 Cádiz, Spain; flavia.amar@uca.es; 3 Virgin of Carmen School, 14001 Cordoba, Spain; 4 Research Group in Sport and Physical Education for Personal and Social Development (GIDEPSO), University of Cordoba, 14071 Cordoba, Spain; m12rocan@uco.es; 5 Research Group in Sport and Physical Education for Personal and Social Development (GIDEPSO), Department of Specific Didactics, Faculty of Education Sciences and Psychology, University of Cordoba, 14071 Cordoba, Spain; m62arcrj@uco.es

**Keywords:** educational physical activity, emotional education, emotional self-regulation, motivation, well-being, pedagogical models

## Abstract

**Background/Objectives**: Emotional intelligence can be understood as the ability to perceive, understand and manage one’s own emotions and those of others, promoting personal and social well-being. In the school context, Physical Education is an ideal setting for developing these skills. The aim of this systematic review was to identify and analyse programmes that integrate emotional intelligence into Physical Education in primary education. **Methods**: To this end, a systematic review was carried out, based on the PRISMA method, in the Web of Science, ERIC and PsycInfo databases, analysing scientific literature related to Physical Education and Emotional Intelligence. Likewise, the PICO strategy was used to develop the inclusion and exclusion criteria, resulting in the selection of 11 articles. **Results**: The results showed that well-planned pedagogical models and active methodologies enable the development of skills such as self-esteem, empathy, emotional self-regulation and motivation. Similarly, integrated approaches that purposefully combine movement and emotion produced more positive and lasting effects than traditional interventions focusing solely on physical aspects. **Conclusions**: The main conclusion is that pedagogical models in Physical Education can promote the development of emotional variables such as empathy, self-regulation, self-confidence, and motivation in primary school students. These findings highlight the need for further research in this area and for the promotion of structured educational programmes that intentionally incorporate emotional work into Physical Education from the early stages of schooling.

## 1. Introduction

Currently, Physical Education must not only focus on students’ motor and academic development but also address their social and emotional dimensions [1]. In this context, emotional intelligence has gained importance as a key factor in psychological wellbeing, academic performance and interpersonal relationships [2,3].

Emotional intelligence can be understood as the ability to perceive, understand, regulate, and use one’s own and others’ emotions effectively [4]. One of its main proponents, Goleman [5], proposed an expanded model identifying five key dimensions: self-awareness, self-regulation, motivation, empathy, and social skills. The accumulation of these skills is essential for effective emotional adaptation in any environment. For his part, Bisquerra [6] emphasises that it should be understood as a personal trait and as a crucial educational skill that must be consciously developed at school.

During primary education, emotions play a significant role and directly influence how children build their personalities, learn to live together and understand the world around them [7]. In this sense, Physical Education, thanks to its experiential, dynamic and playful nature, becomes a perfect setting for working on motor skills and socioemotional competencies such as empathy, self-regulation, self-control and assertiveness [8,9].

In this line, from a neuroscientific perspective, physical activity can promote emotional release and expression. It also considers emotions to be drivers or obstacles to learning. On the other hand, Physical Education understands the body holistically, seeking physical, psychological, and emotional development through motor action. Thus, Physical Education, due to the need to cooperate, compete, develop strategies, or interact with others in an educational context based on physical activity, allows not only for learning related to emotional identification, but also for promoting greater emotional well-being [10,11].

For Physical Education to become a true tool for developing social and personal skills and promoting healthy lifestyles, it must be well structured and planned [12]. To this end, teaching must go beyond physical activity, working on the emotional aspect using the body, with the aim of promoting comprehensive training [13]. In this regard, there are different programmes within Physical Education that have made considerable progress in the emotional intelligence of students. One of them uses the Teaching Personal and Social Responsibility model (TPSR), which has shown a clear improvement in the development of socio-emotional skills, thanks to increased participation, autonomy and respect [14].

Mindfulness-based interventions have proven to be an effective way to improve adaptive emotional regulation [15]. In turn, regular physical activity is associated with an increase in social-emotional skills, provided there is an environment of positive peer relationships [16]. Likewise, Corporal Expression has become an especially valuable tool for working on emotional awareness, self-control and empathy through non-verbal communication, encouraging students to develop new ways of connecting with themselves and others [9].

Similarly, cooperative strategies in Physical Education have proven to be an effective way to strengthen emotional intelligence, promoting skills such as self-control, empathy, and emotional awareness [8]. These skills improve coexistence by creating a more positive environment in the classroom, where students feel more motivated and engaged. In fact, this approach directly influences students’ intrinsic motivation and academic performance [3].

However, despite the value that scientific evidence gives to emotional intelligence in Physical Education, its presence in the curriculum is still limited and, in many cases, disorganised. The reason for this absence is probably due to the fact that Physical Education has focused on content such as strength, endurance, and other more traditional topics, and has neglected content related to the more expressive realm or emotional intelligence, probably due to aspects related to the fact that Physical Education has always been closer to the male model and the gender roles associated with that model [17]. As warned by Benítez-Sillero et al. [12], Physical Education teachers need to receive specific training in methodologies that integrate socio-emotional objectives in a practical and contextualised way.

Therefore, this study is a systematic review whose objective is to identify and analyse the programmes implemented in primary education that address emotional intelligence from the perspective of Physical Education and their impact and improvement on students’ emotional development.

## 2. Materials and Methods

This study aims to conduct a systematic review to identify and analyse existing research on emotional intelligence and Physical Education, especially in primary education. To carry out this review, the guidelines established by the PRISMA statement [18] and the practical guide for systematic reviews with or without meta-analysis [19] were followed. The PICO strategy was used to establish the inclusion and exclusion criteria [20]. Likewise, the procedures were defined prior to the start of the study and subsequently modified, registering the review in PROSPERO (Prospective International Register of Systematic Reviews) with the identification number CRD420251089165, accessible at https://www.crd.york.ac.uk/PROSPERO/view/CRD420251089165 (30 September 2025).

### 2.1. Inclusion Criteria

The inclusion criteria established in this research were: (a) that the study was scientific in nature and addressed the analysis of emotional intelligence, or aspects related to it, from the perspective of Physical Education; (b) that it was a research study; (c) that it focused on primary school students; and (d) that it was written in one of the following languages: Spanish, English or Portuguese (Due to availability and cultural proximity). Likewise, a time frame limited to the last five years (The linguistic and temporal restrictions correspond to the exclusion criteria applied and those works excluded for these reasons have not been considered less relevant) was established with the aim of highlighting the latest research combining aspects related to Emotional Intelligence in Physical Education and presenting a review of the most recent advances, with the aim of presenting and updating the latest research on this topic. The selected articles were the result of a screening process based on these eligibility criteria. In addition, the bibliographic references of the included studies were reviewed to broaden the search. In this regard, to present information with a marked scientific character that meets quality standards, all documents reviewed corresponded to scientific literature, omitting grey literature or non-indexed publications. The time limit set for the search, together with the omission of grey literature, ensured that the publications selected are of high quality, as they were published in high-impact journals. It also ensured that all these studies contain a theoretical basis that corresponds with the literature already available to date.

### 2.2. Search Strategy

The systematic review was conducted in accordance with the guidelines established by the PRISMA statement [18]. To begin the process, a search phrase was defined: (primary education OR primary school OR elementary school OR basic education) AND (Physical education) AND (Emotional intelligence OR Emotional Education OR Emotional skills OR Socio-emotional skills OR Socio-affective skills OR emotional competence OR emotional competency OR emotional competencies OR emotional competencies OR emotional intelligence model OR Emotional learning OR Socio-emotional learning OR Socio-affective learning OR SEL) AND (intervention OR experimental OR quasi-experimental OR randomised controlled trial). Subsequently, articles were searched for in different databases (Web of Science, Psycinfo, and Eric) from 11 April to 16 May 2025. This search was structured into three fundamental areas: (1) Physical Education; (2) emotional intelligence; and (3) intervention, experimental, quasi-experimental, randomised controlled trial. After completing the process, duplicate records were removed.

### 2.3. Study Selection and Data Processing

Once the search process was complete, the titles and abstracts were examined to identify those that adequately met the inclusion criteria, excluding those that did not. As a result, 11 articles were selected and subjected to a detailed analysis, focusing specifically on emotional intelligence as the main topic. The bibliographic references of these works were then reviewed to locate any additional relevant studies; however, no new articles were included. The process was carried out independently by two experts, with discrepancies resolved by consensus. In cases where doubts arose regarding the inclusion or exclusion of a study, a third expert acted to decide on the matter. All three researchers are specialists in the development of systematic reviews and have experience in the field of Physical Education due to their academic training.

**Figure 1:**
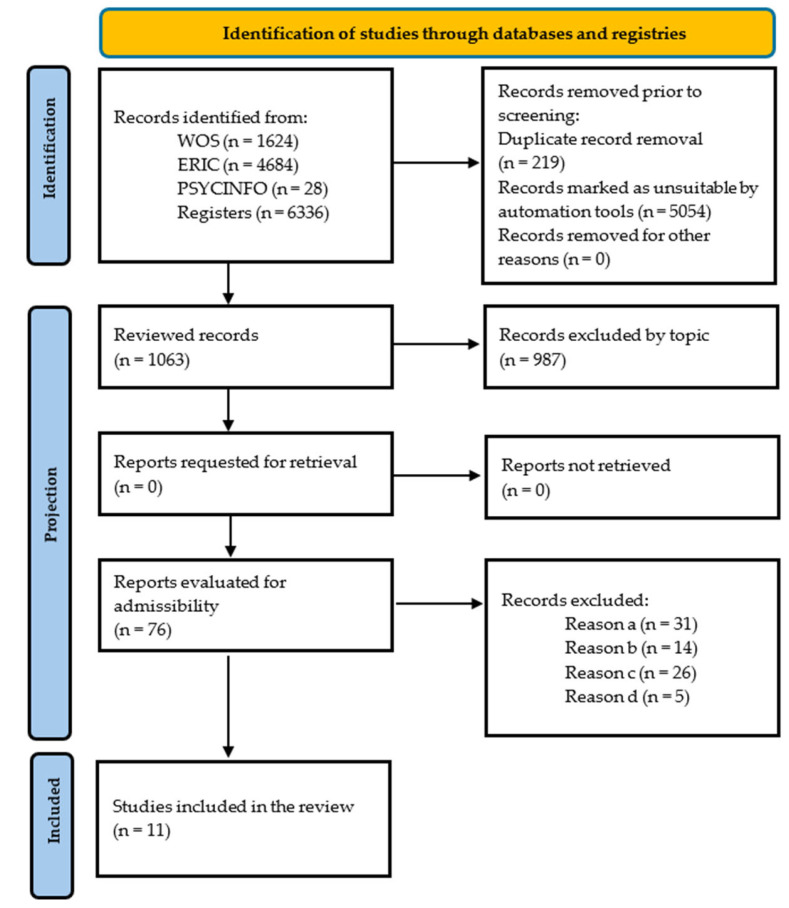
Flowchart (PRISMA, 2020) [18].

Figure 1 shows a flow chart that visually illustrates the procedure followed for selecting the studies included in this review (Figure 1).

### 2.4. Quality Assessment

After selecting the articles included in the review, their quality was assessed using the tool known as “Standard Quality Assessment Criteria for Evaluating Research Papers from a Variety of Fields” [21], designed for quantitative and qualitative studies. Each study was assessed according to the criteria corresponding to each type, considering aspects such as methodological design, sample characteristics, methodological approach, data analysis, and presentation of results and conclusions. The items were rated according to the level of compliance observed: 2 points if it complied satisfactorily, 1 if it complied partially, 0 if it did not comply, and NA if it did not apply (the latter only in quantitative studies). The overall score for quantitative studies was calculated as follows: [(“satisfactory items” × 2) + (“partially satisfactory items” × 1)/28 − (“not applicable items × 2)]. For qualitative studies, the following formula was used: [(“satisfactory items” × 2) + (“unsatisfactory items” × 1)/20]. The results were expressed as a percentage, within a range of 0% to 100%. The evaluation was carried out independently by two researchers (Table S1: Observer assessment 1. Table S2: Observer assessment 2), with the aim of ensuring the greatest possible impartiality. Regarding the assessment of risk of bias, aspects such as selection (studies with non-randomized samples or participant recruitment), performance bias (blinding of informants not clearly identified), and publication bias (omissions of secondary outcomes or protocols not accessible in some cases) can be considered.

### 2.5. Data Collection

First, data were collected from the selected articles. Next, the information collected was verified, following the PRISMA guidelines [18]. The analysis focused on fundamental aspects such as the characteristics of the participants, the type of intervention applied, the results obtained, and the methodological design of each study, all while respecting the structure.

## 3. Results

In the initial phase of the database search, a total of 1063 records were identified, of which 987 were excluded after applying the inclusion and exclusion criteria, also considering the removal of duplicates and those that were not relevant to the topic. As a result, the systematic review finally included a total of 11 articles that met the established requirements (Figure 1). Several manuscripts were excluded for not meeting the inclusion criteria, such as one by Peñalva-Vélez et al. [22].

### 3.1. Quality of Studies

The quality ratings of the articles are represented as percentages, ranging from 0 to 100%, with values between 0.80 and 0.95 (Table 1). To determine the degree of agreement between the evaluators, the intraclass correlation coefficient was used, obtaining a value of 0.791 (*p* = 0.010), which reflects a good degree of agreement [23]. Once the degree of agreement had been established, a conservative inclusion criterion was jointly defined, selecting only those studies with a score of 55% or higher. The overall scores ranged from 0.82 to 0.92 for the first evaluator and from 0.82 to 0.90 for the second.

### 3.2. Study Results

The information extracted from the articles was synthesised according to different units of analysis, which included: Author(s); Country; Context; Subjects; Age; Methodology; Type of study; Duration and Protocol. The main characteristics of the selected studies are presented below (Table 2).

Table 3 summarises the methodologies applied, the instruments used, the variables addressed, and the main findings obtained in the studies analysed. The results highlight the complexity inherent in the process of developing emotional intelligence in the school context, especially in primary education, as well as the role of Physical Education as a means of achieving this.

Based on the research consulted, clear benefits were identified in aspects related to emotional well-being, behavioural commitment, and perception of social competence. These advances occurred in environments where the active participation of students was promoted and they were given space to make decisions autonomously [25,27,28,34]. Programmes that opted for more expressive and creative approaches not only improved students’ emotional state but also helped to build a more inclusive and cooperative classroom environment [25,26].

In addition, the use of active methodologies in Physical Education, such as cooperative learning [24] or gamification [33], was found to be associated with notable improvements in key emotional competencies, such as self-regulation, empathy, self-awareness, and motivation. Taken together, these findings support the need to integrate emotional objectives into traditional classes and reinforce the importance of training teachers in socio-emotional terms. Thus, Physical Education is presented as an effective pedagogical tool for the comprehensive development of students. Likewise, it has been observed that innovations in Physical Education, especially those related to active methodologies and the incorporation of more expressive content, are more suitable for developing emotional skills in students.

In terms of the duration and effectiveness of the program, it can be observed that longer programs achieve more solid and significant results [24,27] than shorter ones [29,34].

In relation to gender, heterogeneity is observed in terms of differences, with improvements in boys in interventions based on Corporal Expression [26] and an increase in negative emotions in more competitive contexts [29]. In relation to girls, these changes are less intense [26].

In terms of context, those that incorporated variables related to social skills, social responsibility, or cooperation showed positive results in more dimensions related to SEL [24,28,30].

On the other hand, it could be considered that the risk of bias in the reports included could be moderate overall, finding studies that do not have registered or accessible protocols. The results should be considered with caution due to the diversity of thoroughness in the presentation of neutral or negative results. Similarly, the certainty of evidence should be considered with caution due to the heterogeneity in the results, instruments, sample size, or type of design of the studies analysed.

Table 4 below presents the main findings regarding the authors and duration of the programs, the bases for intervention, and the social-emotional dimensions addressed.

Table 4 defines the extent to which emotional intelligence advances depending on the basis of the intervention. In this sense, expressive methodologies show progress in a greater number of dimensions than the other bases of intervention. Thus, they stand out for presenting dimensions such as emotional expressiveness or the reduction of aggressive behaviour, which do not appear in other cases [25,26].

Active methodologies stand out for presenting dimensions such as autonomy, cooperation, conflict resolution, and participation [24,28,32,33,34]. They also coincide with expressive methodologies in dimensions such as respect, motivation, and self-esteem [25,26]. In this line, gamification is presented as more effective and closer to emotional intelligence models [33].

Extracurricular physical activity promotes improvements in motivation and social skills [30], coinciding with expressive [25,26] and active methodologies [24,28,32,33,34], as well as improvements in SEL. Improvements are also seen in aspects related to mental health, in this case through methodologies that seek relaxation and body awareness [27]. In this sense, the results point to differences in the improvement of dimensions depending on the methodologies implemented.

## 4. Discussion

The objective of this systematic review was to identify and analyse programmes implemented in primary education that address emotional intelligence from the perspective of Physical Education and their impact and improvement on students’ emotional development.

After analysing the results obtained, the value of emotional intelligence in the comprehensive development of students becomes evident, as does how Physical Education can become an ideal educational space to work on it. Theoretical approaches [4,5,6] had already highlighted that emotional intelligence directly influences well-being, coexistence and academic performance. With the aim of reinforcing this idea, there are diverse types of methodology that, when applied in Physical Education, positively and lastingly enhance the dimensions of emotional intelligence.

Specifically, improvements can be observed in skills such as self-control, self-esteem and emotional awareness in interventions that used expressive methodologies, such as dramatisation or body language [25,26]. Aguilar-Herrero [49] demonstrates through the application of the “Move Against Bullying” programme that skills such as empathy, self-regulation and group cohesion can be promoted through the use of Corporal Expression, supporting the idea that Physical Education can influence emotional competence and help prevent school violence. According to Armada-Crespo [35], Corporal Expression is a component of Physical Education that acts to channel emotions, reinforce personal identity and build more harmonious coexistence, clearly influencing the comprehensive education of students.

For their part, programmes that incorporated active methodologies, such as cooperative learning [24] or the TPSR model [28,34], achieved clear improvements in such key areas as empathy, leadership skills, responsibility and cooperation. These advances coincide with the findings of Rivera-Pérez et al. [8] and Gil-Moreno & Rico-González [3] who emphasise that collaboration and group work strengthen socio-emotional skills that, in addition to being useful in school, are essential for positive coexistence and a more balanced environment.

Furthermore, the emotional benefits were particularly evident in well-designed sessions. Kliziene et al. [27] observed a significant reduction in anxiety among Physical Education students when classes were structured with intention and care. This finding underscores the importance of planning with a clear purpose, seeking to promote emotional well-being and physical performance.

From another perspective, recent research such as that by Carcelén-Fraile [33] and Fenalampir et al. [32], who advocate the use of innovative methodologies such as gamification or the HPC approach, demonstrates benefits in dimensions of children’s socio-emotional development such as motivation, self-confidence, empathy, and emotional self-regulation. These conclusions are in line with studies such as that by Wright et al. [50] who argue that meaningful and motivating methodologies have the power to engage students and enhance their emotional growth in the classroom.

Similarly, the results of Goh et al. [30] and Melguizo-Ibañez et al. [31] support the idea that systematic physical activity, both inside and outside school hours, is associated with an increase in socio-emotional skills. Specifically, improvements were observed in self-esteem, self-concept and emotional repair, which coincides with the contributions of Wang et al. [16] highlighting the relationship between regular exercise and socio-emotional skills, especially in contexts where interpersonal relationships between peers are positive. These effects reinforce the view of Gordon et al. [13] who defend the comprehensive educational value of Physical Education when it is oriented towards goals beyond physical performance.

In this regard, it can be observed that studies that develop interventions based on expressive techniques such as Corporal Expression or Dramatization [25,26] show significant improvements in a greater number of dimensions of personal and social development than interventions that focus on methodological aspects [24,33], suggesting that expressive content may be relevant in the development of emotional intelligence. Likewise, in terms of duration and effectiveness, those interventions with a longer duration presented more significant results than those with fewer hours.

In this sense, expressive methodologies and active methodologies show more significant improvements than interventions based on relaxation and body awareness or extracurricular physical activity. Thus, expressive methodologies seem to enhance aspects related to the individual (emotional expressiveness, self-esteem, self-control, motivation) and in relation to others (social skills, respect, or reduction of aggressive behaviour) [25,26]. This connects with active methodologies such as cooperative learning, gamification, or TPSR. In this way, gamification coincides with expressive methodologies in aspects related to the individual (self-esteem and self-concept) and in relation to peers (social skills) [33]. On the other hand, cooperative learning refers to improvements in dimensions that connect with others (empathy, respect, and cooperation) [24]. This is repeated in an equivalent way with TPSR (participation and conflict resolution), showing some improvements in the personal sphere (autonomy and responsibility) [24,28,32,33,34].

In contrast, more traditional approaches tend to leave the emotional component in the background. As a result, they generate competitive dynamics that can provoke negative emotions such as frustration, anxiety, or demotivation, especially among students with lower motor skills or fragile self-esteem [29]. In this line, the use of different methodologies has an impact on the dimensions of emotional intelligence that improve.

The findings obtained in this systematic review demonstrate that Physical Education interventions could positively influence the five dimensions of emotional intelligence [5], thus reinforcing the value of the emotional approach in Physical Education as a vehicle for achieving comprehensive education [4,6,10,11]. The connection between the results and the theoretical frameworks reviewed suggests that Physical Education, when designed from an emotional perspective, could act as a privileged educational space for SEL. In line with previous findings and literature, it is necessary to specifically incorporate emotional intelligence into education laws and curricula for the training of future Physical Education teachers. This would involve improving this subject based on existing premises and aligned with the true nature of the activity proposed by fields such as neuroscience. Building the subject on scientific evidence will improve the quality of Physical Education, in line with the 4th sustainable development goal (Quality Education).

However, despite the positive results, some limitations found in the reviewed studies must also be addressed. These limitations could be differentiated between methodological and review limitations. First, some of them have methodological shortcomings, such as the use of small samples or the absence of control groups, which complicates the possibility of applying these findings to other educational contexts with confidence. Furthermore, most studies follow quantitative approaches, leaving aside the more experiential part of the students’ experience. This limits the ability to understand how students live, feel, or interpret these interventions. Therefore, incorporating mixed methods could enrich the research and generate a more comprehensive and closer approach. There is also variability between the methodologies used, making it difficult to compare results directly and draw generalisable conclusions. On the other hand, and taking into account the limitations related to the review itself, all the studies focus solely on primary education, without considering the effects that these interventions could have at other stages of education. There is also a language bias, as most of the studies are written in Spanish, which can make it difficult to access relevant research published in other languages. On the other hand, several challenges can be observed in teaching planning, since certain methodologies, such as those focused on competition, could induce negative emotions such as frustration, anger or sadness. Therefore, for good teaching practice, it is necessary to provide teachers with continuous training in content related to emotional intelligence [12].

Given these limitations, future lines of research should focus on designing studies with more rigorous and systematic methodologies and larger and more diverse samples that examine the dose–response effect more systematically. Similarly, it would be advisable to develop mixed-method research that analyses students’ experiences of the programmes implemented. It is also considered essential to broaden the focus to other educational stages to evaluate the impact of the proposals in contexts other than primary education. In turn, it would be interesting to review studies published in other languages, thereby broadening the theoretical and empirical framework. Another fundamental aspect is to investigate how teachers’ knowledge of emotional intelligence influences the planning of sessions and the creation of positive affective climates within the classroom. Finally, technology could be key to opening a wide range of possibilities for working on emotional intelligence in a more motivating and accessible way that is adapted to the needs of students.

## 5. Conclusions

The results of this systematic review allow us to conclude that Physical Education can be an ideal space for both the development of motor skills and the promotion of emotional intelligence in primary school. The evidence collected shows that, thanks to active and well-planned methodologies, emotional variables such as empathy, self-regulation, self-confidence and motivation can be significantly improved. Furthermore, by intentionally incorporating socio-emotional content into this subject, teaching practice could be enriched, and the psychosocial well-being of students could be affected. Likewise, expressive content promotes the improvement of emotional intelligence in primary school students. For all these reasons, research in Physical Education in relation to Emotional Intelligence in Primary Education should take into account the use of Active Methodologies, the use of Corporal Expression and, above all, rigorous and exhaustive planning that allows for the implementation of programs that positively influence students’ social-emotional skills. Likewise, the use of mixed methods should be considered, as they allow for more complete information to be gathered on the students’ experience, as well as broader and more diverse samples. Considering the scientific evidence and findings, Physical Education should include a curriculum, training and practical application in schools that addresses not only motor skills, but also psychological and emotional aspects. Thus, one of the main innovations presented in this systematic review is the review of Physical Education from a perspective that goes beyond the physical aspect alone. This allows the subject to be understood as holistic content that enables the comprehensive development of students. For all these reasons, future research should overcome the methodological limitations presented above and develop actions that combine methodologies to have a significant impact on a greater number of dimensions. This would mean focusing Physical Education on progress in relation to motor skills, but without neglecting psychological and emotional development.

This study presents a variety of practical applications in the field of education, specifically aimed at primary school teachers. On the one hand, it provides a systematic and up-to-date review of emotional intelligence in Physical Education. On the other hand, it includes specific teaching strategies for working on these skills in the classroom. For emotionally competent planning, the resources collected in the review can serve as a guide for teacher training, contributing to the construction of a more affective, inclusive and motivating environment. In short, this research aims to provide a solid foundation that enriches teaching from an emotional point of view, with clear proposals for which practices yield results in real contexts and how to adapt them to the needs of the classroom.

## Figures and Tables

**Table 1:** Quality ratings of articles.

Studies	Observer 1	Observer 2
Bjorke & Moen (2020) [24]	0.90	0.90
Aguilar et al. (2021) [25]	0.85	0.82
Castillo et al. (2021) [26]	0.89	0.85
Kliziene et al. (2021) [27]	0.82	0.82
Simonton & Shiver (2021) [28]	0.92	0.90
Álvarez & Fernández (2022) [29]	0.85	0.85
Goh et al. (2022) [30]	0.92	0.90
Melguizo et al. (2022) [31]	0.85	0.85
Fenanlampir et al. (2024) [32]	0.89	0.85
Carcelén-Fraile et al. (2025) [33]	0.89	0.85
Sindiani et al. (2025) [34]	0.85	0.90

**Table 2:** Main Characteristics of the Study Sample.

Authors	Country	Context	Subjects	Age	Methodology	Type of Study	Duration	Protocol	
								Control Group	Experimental Group
Bjørke & Mordal Moen (2020) [24]	Norway	Primary education	64	10 to 12 years	Qualitative	Action research	24 weeks spread over 2 years	NCG	All subjects participated in the cooperative learning model using different methods (Jigsaw, Think-Pair-Share, etc.)
Aguilar Herrero et al. (2021) [25]	Spain	Primary education	29 (males) and 29 (females)	10 to 12 years	Quantitative	Quasi-experimental	6 weeks (2 h per week)	NCG	The teaching unit consisted of sessions associated with body language, with the aim of raising awareness of one’s own and others’ emotions and regulating them.
Castillo-Viera et al. (2020) [26]	Spain	Primary education	141 (boys) and 153 (girls)	11 and 12 years	Quantitative	Quasi-experimental	8 weeks (twice a week)	NCG	An intervention programme based on the content block of body expression, specifically dramatisation, was implemented.
Kliziene et al. (2021) [27]	Lithuania	Primary education	364 186 experimental group 90 (males) and 96 (females) 181 control group 91 (males) and 90 (females)	6 to 9 years	Quantitative	Experimental	8 months (three times per week)	Attended regular Physical Education classes, without modifications or special intervention	Received a structured programme with a methodology based on the DIDSFA model, organising classes into thematic blocks and combining physical abilities, motor skills and relaxation, concentration and reflection activities
Simonton & Shiver (2021) [28]	United States	Primary education	124 (males) and 98 (females)	7 to 9 years	Quantitative	Comparative and correlational, with a cross-sectional design	9 months (twice a week, 35-min classes)	Received regular Physical Education classes	Participated in classes organised according to the TPSR, consisting of motor tasks integrated with responsibility objectives through a focus on the development of social and emotional skills
Álvarez-Ibáñez & Fernández-Hawrylak (2022) [29]	Spain	Primary education	43 21 experimental group 9 (men) and 12 (women) 22 control group 10 (males) and 12 (females)	11 and 12 years	Quantitative	Quasi-experimental	2 h	Completed a pre- and post-test questionnaire and participated in a non-competitive session	Completed a pre- and post-test questionnaire and participated in a competitive session
Goh et al. (2022) [30]	United States	Primary education	138 75 experimental group (35 males and 40 females) 63 control group (28 males and 35 females)	9 to 13 years	Quantitative	Quasi-experimental	6 weeks (3 times per week)	Did not receive any intervention	Participated in a pre-school physical activity programme, with 30 min sessions including warm-up activities, fitness exercises, team sports and games, and relaxation, to promote social and emotional competence
Melguizo-Ibáñez et al. (2022) [31]	Spain	Primary education	303 (boys) and 264 (girls)	9 to 13 years	Quantitative	Non-experimental (ex post facto), descriptive and cross-sectional	NR	NCG	Two groups were compared: (a) physically active group (more than 3 h of physical activity per week) and (b) less active group (less than 3 h of physical activity per week) by completing a questionnaire related to emotional intelligence, body mass index and adherence to the Mediterranean diet.
Fenanlampir et al. (2024) [32]	Indonesia	Primary education	90 47 (males) and 43 (females)	9 to 12 years	Quantitative	Quasi-experimental	NR	NCG	Received an emotional skills test after applying the HPC (Homogeneity Psycho Cognition) strategy, as well as a questionnaire comparing their conventional classes with the classes where the strategy was applied
Carcelén-Fraile (2025) [33]	Spain	Primary Education	120 60 experimental group 60 control group	8 to 11 years	Quantitative	Randomised controlled trial	3 months (3 times a week)	Continued with their usual Physical Education classes to achieve physical goals through functional or technical activities	Received a programme that integrated active gamification into physical activity, transforming the sessions into motivating and immersive experiences through progressive narratives, point systems, rewards, etc.
Sindiani et al. (2025) [34]	Israel	Primary education	260 185 experimental group 75 control group	9 to 12 years	Mixed	Experimental research with a mixed convergent design	3 sessions of 45 min	Did not receive any intervention	A social-emotional learning (SEL) programme based on the TPSR model focused on cooperative games was implemented

NR: No reported. NCG: No Control Group.

**Table 3:** Treatment Variables and Main Results and Relationships of Physical Education with Emotional Intelligence.

Studies	Objectives	Variables	Instruments	Main Results
Bjørke & Mordal Moen (2020) [24]	To analyse the impact of a programme based on cooperative learning on the development of the subjects’ social and emotional skills	Attitude towards cooperative learning Social and emotional skills Understanding PE as a learning subject	Interviews with teachers and students (before, during and after Recorded professional development workshops Systematic observations Researcher’s reflective journal Post-lesson analysis	Students showed a positive change in their attitude towards cooperative learning. In addition, improvements were observed in their social and emotional skills, developing aspects such as empathy, respect and the ability to work in a team
Aguilar Herrero et al. (2021) [25]	Examine the outcome of implementing an educational programme based on Body Expression during Physical Education classes to promote social-emotional skills and prevent bullying.	Physical activity Empathy Assertiveness Social relationships Cooperation and leadership Respect and responsibility Respect and responsibility Conflict resolution Emotional management Self-esteem Victimisation-aggression	Socio-affective skills questionnaire [35] Sociometric questionnaire	Significant improvements were observed in some dimensions, especially in social relationships, respect and responsibility, as well as in self-esteem and the reduction of behaviours related to victimisation and aggression. However, no relevant changes were observed in other areas such as assertiveness, emotional management or conflict resolution.
Castillo-Viera et al. (2020) [26]	Investigating the effect of a drama programme in Physical Education classes on the development of emotional intelligence in 6th grade primary school students	Physical activity Emotional expressiveness Self-control Motivation Self-awareness Social skills	Emotional intelligence questionnaire for children [36]	The experimental group showed significant improvements in aspects related to emotional expressiveness, self-control, motivation and self-awareness, as well as a slight improvement in their social skills. In contrast, the control group did not show any significant changes. It should be noted that the improvements were particularly noticeable in boys.
Kliziene et al. (2021) [27]	To evaluate the effect of a Physical Education programme on physical activity and emotional well-being, specifically on anxiety, in primary school children.	Physical activity Emotional anxiety	Children’s Physical Activity Questionnaire (C-PAQ) Revised Children’s Manifest Anxiety Scale (RCMAS)	The experimental group showed a significant increase in physical activity, measured in MET units, compared to the control group. Likewise, a reduction in anxiety levels was observed, both in its somatic dimension and in the components of personality and social anxiety. These effects were evident in children between the ages of 8 and 9.
Simonton & Shiver (2021) [28]	To explore the relationship between students’ emotions in PE classes and their behaviours related to attitude and responsibility, by comparing the effects of a traditional curriculum with one based on the TPSR.	Physical activity Emotions (enjoyment, boredom, and anger) Behaviour (perceived responsibility, commitment, problems with school functioning)	Achievement Emotions Questionnaire—Elementary School (AEQ-ES) [37], adapted to Physical Education Subscale from the social-goal scale [38] Behavioural commitment subscale from [39] Subscale from the paediatric quality of life inventory (PedsQL) [40]	Enjoyment was shown to be a significant predictor of behavioural commitment despite not showing relevant relationships with other behavioural variables. In contrast, anger acted as a significant negative predictor in the three outcomes analysed. However, boredom did not prove to be a significant predictor. In general terms, the experimental group showed higher levels of enjoyment, responsibility and commitment, as well as lower levels of boredom, anger and school problems compared to the control group
Álvarez-Ibáñez & Fernández-Hawrylak (2022) [29]	Comparing the emotional impact of competitive versus non-competitive physical activity on primary school students in Physical Education classes	Physical activity (competitive and non-competitive) Emotional states	POMS (Profile of Mood States) questionnaire adapted to the subjects [41,42]	A greater presence of negative emotions, such as anger and depression, was observed in the group that participated in competitive physical activities. In contrast, no significant differences were found between the groups in the dimensions of vigour, tension or fatigue
Goh et al. (2022) [30]	To assess the evolution of social and emotional learning (SEL) competence in primary and secondary school students participating in a physical activity programme before the start of classes	Physical activity SEL competence	Adapted DESSA-Mini questionnaire	The experimental group experienced a significant 7% increase in scores related to SEL competencies after the programme was implemented, while the control group showed no significant changes. No significant differences were found based on gender. In addition, the programme contributed positively to improving interaction among students, enhancing their motivation, and promoting the development of relational skills.
Melguizo-Ibáñez et al. (2022) [31]	To study the relationship between emotional intelligence, adherence to the Mediterranean diet, Body Mass Index (BMI) and age, considering whether students engage in more or less than 3 h of physical activity per week.	Physical activity Age Emotional intelligence Mediterranean diet BMI	TMMS-24 [43,44] KIDMED questionnaire BMI calculation	Students who engaged in more than 3 h of physical activity per week showed higher levels of emotional intelligence. A particularly interesting finding was the positive relationship between emotional repair ability and greater adherence to a healthy diet. On the other hand, an inverse relationship was detected between BMI and emotional repair ability.
Fenanlampir et al. (2024) [32]	Analysing the effect of the Homogeneity Psycho Cognition (HPC) learning strategy on the emotional skills of primary school students in Physical Education classes	Physical activity HPC Emotional skills (self-awareness, self-regulation, empathy, regulation, and social skills)	Emotional skills questionnaire (based on Goleman [45]) Statistical test (ANOVA and LSD test) Observation sheet and response questionnaire	The experimental group obtained higher scores in emotional skills compared to the control group. Specifically, the HPC programme had a positive impact in areas such as self-confidence, motivation, empathy and personal autonomy.
Carcelén-Fraile (2025) [33]	Determining the effectiveness of an active gamification programme on emotional well-being and social skills in primary school students	Physical activity Self-esteem Self-concept Social skills	School Self-Esteem Test [46] Garley Self-Concept Questionnaire (CAG) MESSY Questionnaire [47]; Spanish version: [48]	The experimental group showed a clear improvement in self-esteem and in all dimensions of self-concept. Progress was also observed in the development of social skills, along with a reduction in impulsive behaviours and overconfident attitudes. However, no significant differences were detected in aspects such as jealousy or feelings of loneliness.
Sindiani et al. (2025) [34]	To examine the effectiveness of a social and emotional learning (SEL) programme implemented during Physical Education classes in primary school, with a special focus on teamwork skills, self-awareness and creative thinking	Physical activity Teamwork Self-awareness Team thinking	Systematic observations with a 1–4 scale rubric Semi-structured interviews	The experimental group showed significant improvements in the three skills assessed, with particularly marked progress among those students who initially participated less, suggesting a positive impact of the programme. In addition, a change was observed in the teachers’ perspective, i.e., they began to value not only physical abilities but also emotional and social skills. As a result, they began to give them more autonomy and space to make decisions.

**Table 4:** Study basis and improved dimensions.

Studies/ Duration	Bases for Intervention	Improved Dimensions
	**Expressive methodologies**	
Aguilar Herrero et al. (2021) [25] 6 weeks	Dramatisation	Emotional expressiveness Motivation Self-control Social skills
Castillo-Viera et al. (2020) [26] 8 weeks	Corporal Expression	Social skills Self-esteem Respect Reduction in aggressive behaviour
	**Active methodologies**	
Bjørke & Mordal Moen (2020) [24] 24 weeks (2 years)	Cooperative learning Empathy, respect, and teamwork	Empathy Respect Cooperation
Sindiani et al. (2025) [34] 3 lessons	TPSR	Participation Autonomy
Simonton & Shiver (2021) [28] 9 months	TPSR	Responsibility Conflict resolution
Carcelén-Fraile (2025) [33] 3 months	Gamification	Self-esteem Self-concept Social skills
Fenanlampir et al. (2024) [32] NR	Homogeneity Psycho Cognition strategy	Autonomy Empathy Motivation Self-confidence
Álvarez-Ibáñez & Fernández-Hawrylak (2022) [29] 2 h	Competitive versus non-competitive physical activity programme	Competitive ⟶ negative attitudes. Non-competitive ⟶ positive emotional experiences
	**Extracurricular physical activity**	
Goh et al. (2022) [30] 6 weeks	Physical activity programme before the start of classes	Improvements in SEL Motivation Social skills
Melguizo-Ibáñez et al. (2022) [31] NR	Relationship between emotional intelligence, healthy lifestyle habits and physical activity	Higher level of emotional intelligence and healthier lifestyle habits in students who engaged in more than three hours of physical activity per week
	**Relaxation and body awareness**	
Kliziene et al. (2021) [27] 8 months	DIDSFA model	Reduction in anxiety levels

## Data Availability

All information relating to the results can be found in the article or in Supplementary Materials. For further information, please consult the bibliographical references of the articles selected for the study.

## Supplementary Materials

The following supporting information can be downloaded at: https://www.mdpi.com/article/10.3390/healthcare13233166/s1, Table S1: Observer assessment 1. Table S2: Observer assessment 2.

## Abbreviations

The following abbreviations are used in this manuscript:

PROSPERO	Prospective International Register of Systematic Reviews
TPSR	Teaching Personal and Social Responsibility
SEL	Social-emotional learning
BMI	Body Mass Index
HPC	Homogeneity Psycho Cognition
NR	No reported
NCG	No control group
