# Supplementary material for: Emotional Intelligence in Physical Education in Primary Education: A Systematic Review
Source: Healthcare (Basel). 2025 Dec 3;13(23):3166. doi: 10.3390/healthcare13233166 (PMC12691935; doi:10.3390/healthcare13233166)
Supplement: Supplementary file 1 [file healthcare-13-03166-s001.zip › Table S2 - Observer 2.pdf]

Observer 2

Studies	1	2	3	4	5	6	7	8	9	10	11	12	13	14	Results
Bjorke & Moen (2020)	2	2	2	2	1	1	2	2	2	2					0.9
Aguilar et al. (2021)	2	2	2	2	2	2	2	0	2	2	1	1	1	2	0.82
Castillo et al. (2021)	2	2	2	2	2	1	2	2	2	1	2	0	2	2	0.85
Kliziene et al. (2021)	2	2	1	2	1	1	2	2	1	2	1	1	2	2	0.82
Simonton & Shiver (2021)	2	2	2	2	2	2	2	2	1	2	2	0	2	2	0.90
Álvarez & Fernández (2022)	2	2	1	2	2	2	2	2	2	2	2	0	1	2	0.85
Goh et al. (2022)	2	2	2	2	2	2	2	2	1	2	2	0	2	2	0.90
Melguizo et al. (2022)	2	2	2	2	1	2	2	2	1	2	2	0	2	2	0.85
Fenanlampir et al. (2024)	2	1	2	2	1	2	2	2	2	2	2	0	2	2	0.85
Carcelén-Fraile et al. (2025)	2	2	2	2	2	2	2	2	1	2	1	0	2	2	0.85
Sindiani et al. (2025)	2	2	2	2	1	2	1	2	2	2					0.90
